# COVID-19-Impfintention von Eltern bezogen auf ihre Kinder

**DOI:** 10.1007/s00103-022-03613-z

**Published:** 2022-11-09

**Authors:** Susanne Brandstetter, Maja Pawellek, Merle M. Böhmer, Angela Köninger, Michael Melter, Michael Kabesch, Christian Apfelbacher, KUNO-Kids Study Group

**Affiliations:** 1 KinderUniKlinik Ostbayern (KUNO), Lehrstuhl für Kinder- und Jugendmedizin der Universität Regensburg, Regensburg, Deutschland; 2 Wissenschafts- und Entwicklungscampus Regensburg (WECARE), Klinik St. Hedwig, Barmherzige Brüder, Regensburg, Deutschland; 3 Bayerisches Landesamt für Gesundheit und Lebensmittelsicherheit (LGL), München, Deutschland; 4 Institut für Sozialmedizin und Gesundheitssystemforschung, Otto-von-Guericke-Universität Magdeburg, Leipziger Straße 44, 39120 Magdeburg, Deutschland; 5 Klinik für Geburtshilfe und Frauenheilkunde der Universität Regensburg, Klinik St. Hedwig, Regensburg, Deutschland

**Keywords:** COVID-19, Impfung, Impfintention, Impfbereitschaft, Kinder, COVID-19, Vaccination, Intention to vaccinate, Willingness to vaccinate, Children

## Abstract

**Hintergrund:**

Mit Beginn der Impfkampagne hat eine neue Phase der Bewältigung der Coronapandemie begonnen. Zulassung und Empfehlung für die COVID-19-Impfung von Kindern folgten schrittweise; bis heute (mit Stand vom 04.10.2022) sind in Deutschland Impfungen für Kinder unter 5 Jahren nicht zugelassen.

**Ziel der Arbeit:**

Ziel war es, zu untersuchen, wie sich die COVID-19-Impfintention von Eltern bezogen auf ihre Kinder von Mai 2020 bis Februar 2021 (von der ersten zur zweiten Coronawelle) entwickelte und welche Determinanten der Impfintention es gibt.

**Methoden:**

612 Familien, die mit ihrem Kind im Alter zwischen 1,5 und 6 Jahren an der KUNO Kids Gesundheitsstudie teilnehmen, beantworteten im Mai 2020 Fragen eines Online-Surveys (Teilnahmerate 51 %), 507 nahmen an der Wiederholungsbefragung im Februar 2021 teil. Determinanten der Impfintention wurden für beide Zeitpunkte in uni- und multivariablen logistischen Regressionsmodellen analysiert.

**Ergebnisse:**

Während 51 % der Eltern im Mai 2020 angaben, ihr Kind gegen COVID-19 impfen lassen zu wollen, reduzierte sich dieser Anteil bis Februar 2021 auf 41 %. Zu mindestens einem der beiden Zeitpunkte waren Gesundheitskompetenz sowie die wahrgenommene Kompetenz bzgl. Schutzmaßnahmen gegen das Virus signifikant positiv mit einer höheren Impfintention assoziiert, die Zugehörigkeit von Angehörigen zu einer Risikogruppe sowie der Eindruck, dass die politischen Maßnahmen übertrieben seien, ging mit einer niedrigeren Impfintention einher.

**Diskussion:**

Die Intention, das Kind gegen COVID-19 impfen zu lassen, war nur mäßig ausgeprägt und nahm zur zweiten Coronawelle weiter ab. Einstellungs- und kompetenzbezogene Determinanten waren zu beiden Zeitpunkten wichtig und könnten in einer künftigen Impfkampagne, die Eltern jüngerer Kinder adressiert, gezielt berücksichtigt werden.

**Zusatzmaterial online:**

Zusätzliche Informationen sind in der Online-Version dieses Artikels (10.1007/s00103-022-03613-z) enthalten.

## Abstract (en)

**Background:**

With the start of the vaccination campaign, a new phase in the management of the coronavirus pandemic has begun. Approval and recommendation for COVID-19 vaccination of children followed gradually; to date (4 October 2022), vaccination for children under five years of age has not been approved in Germany.

**Aim of the study:**

The aim was to investigate how parents’ intention to vaccinate their children against COVID-19 developed from May 2020 to February 2021 (from the first to the second wave of the COVID-19 pandemic) and to analyse the determinants of the intention to vaccinate.

**Methods:**

In May 2020, 612 families participating with their children aged 1.5–6 years in the KUNO Kids Health Study completed an online survey (participation rate 51%), and 507 completed the second survey in February 2021. Determinants of the intention to vaccinate were analysed for both time points using univariable and multivariable logistic regression models.

**Results:**

While 51% of parents reported wanting their children vaccinated against COVID-19 in May 2020, this proportion decreased to 41% by February 2021. At least at one of the two time points, health literacy and perceived competence regarding protective measures against the virus were significantly positively associated with higher vaccination intentions, while belonging to a risk group and the perception that the political measures were exaggerated were associated with lower vaccination intentions.

**Discussion:**

Parents’ intention to have their children vaccinated against COVID-19 was low and decreased further from the first to the second wave of the coronavirus pandemic. Attitudinal and competence-related determinants were important at both time points and could be targeted in a future vaccination campaign addressing parents of younger children.

## Einleitung

Die öffentliche Wahrnehmung der COVID-(Coronavirus-Disease-)19-Pandemie ist von einem wellenartig auftretenden Infektionsgeschehen sowie von Fortschritten in den Maßnahmen zur Pandemiebewältigung geprägt. Mit der Verfügbarkeit sicherer und effektiver Impfstoffe begann eine neue Phase. Die Impfkampagne zur COVID-19-Impfung in Deutschland begann im Dezember 2020 mit der Impfeinladung für Erwachsene; erst im August 2021 wurde die Impfempfehlung für Jugendliche ab 12 Jahren ausgesprochen. Seit Dezember 2021 ist die COVID-19-Impfung auch für Kinder im Alter von 5–11 Jahren zugelassen [1], zunächst jedoch nur mit einer eingeschränkten Empfehlung: Anfangs hatte die Ständige Impfkommission (STIKO) am Robert Koch-Institut (RKI) für diese Altersgruppe keine allgemeine Impfempfehlung ausgesprochen, sondern empfahl die Impfung lediglich den Kindern, die wegen Vorerkrankungen ein höheres Risiko für einen schweren Verlauf nach einer Infektion mit dem neuartigen Coronavirus (SARS-CoV-2) haben, bzw. Kindern, deren Angehörige zu einer Risikogruppe gehören und selbst nicht geimpft werden können. Im Mai 2022 folgte schließlich die allgemeine Impfempfehlung für die 5‑ bis 11-jährigen Kinder [2]. Ob und wann es eine Ausweitung der Impfempfehlung auch für jüngere Kinder geben wird, ist bislang nicht absehbar.

Die Bedeutung der COVID-19-Impfung für Kinder wurde und wird kontrovers diskutiert. Der Diskurs ist einerseits geprägt von dem Argument, dass Kinder und Jugendliche selbst in der überwiegenden Anzahl der Fälle nicht oder nur leicht erkranken und ihr Beitrag zum Erreichen der Herdenimmunität fraglich sei – insbesondere im Lichte nur unzulänglicher Impfquoten unter Erwachsenen [3]. Andererseits besteht die Sorge vor den Folgen einer SARS-CoV-2-Infektion: Das Paediatric Inflammatory Multisystem Syndrome (PIMS-TS; [4, 5]) sowie das Post-COVID-19-Syndrom [6] gehen mit schwerwiegenden Beeinträchtigungen einher, welche durch einen Schutz vor einer Infektion verhindert werden könnten.

Skepsis von Eltern gegenüber Impfungen bei ihren Kindern ist – insbesondere in westlichen Ländern – ein verbreitetes Phänomen [7, 8]. Daten der für Kinder und Jugendliche in Deutschland repräsentativen KiGGS-Studie zeigen, dass sich die Durchimpfungsrate bezüglich der Standardimpfungen im Laufe von 10 Jahren verbessert, bei den 3‑ bis 6‑jährigen Kindern bisher jedoch nicht die angestrebte Quote von 95 % erreicht wurde [9]. Die Weltgesundheitsorganisation (WHO) betrachtet Impfmüdigkeit („vaccine hesitancy“) als eine der 10 Bedrohungen für die globale Gesundheit [10]. Mit Impfmüdigkeit wird beschrieben, dass es ein Kontinuum zwischen Ablehnung und Akzeptanz von Impfungen gibt, auf dem sich Individuen unterschiedlich verorten [11]. Ob eine Impfung eher akzeptiert oder abgelehnt wird, hängt dabei von individuellen einstellungsbezogenen Faktoren ab. Dazu gehören u. a. Abwägungen zum persönlichen Risiko bzw. Nutzen durch die Impfung oder das Vertrauen in die Sicherheit des Impfstoffs und in staatliche Organisationen. Häufig kommen in Studien zu Determinanten von Akzeptanz oder Ablehnung von Impfungen gesundheitspsychologische Verhaltensmodelle zur Anwendung. Ein Ansatz, der verschiedene Theorien integriert, ist das 5C-Modell [12, 13]. Gründe für oder gegen Impfungen werden hier in den Domänen Vertrauen („confidence“), Risikowahrnehmung („complacency“), wahrgenommene praktische Barrieren („constraints“), Informationssuche („calculation“) und kollektive Verantwortung („collective responsibility“) gefasst. Diese individuellen psychologischen Faktoren sind eingebettet in politische und soziokulturelle Kontextfaktoren.

Vor dem Hintergrund der Coronapandemie stellt sich nun die Frage, wie viele Eltern eine COVID-19-Impfung für ihr Kind beabsichtigen. Aus bisherigen Studien gibt es Hinweise, dass die Impfbereitschaft insbesondere für jüngere Kinder nur mäßig ausgeprägt ist [14]. Die sich rasch wandelnde pandemische Lage macht es zudem erforderlich, mögliche Veränderungen der elterlichen Impfintention im Verlauf der Coronapandemie zu beschreiben und Faktoren zu identifizieren, die die elterliche Impfentscheidung beeinflussen. Dies kann helfen, aktuelle und künftige Impfkampagnen zu gestalten und zu steuern.

Ziel der vorliegenden Arbeit war es daher, zu untersuchen, wie sich die COVID-19-Impfintention von Eltern bezogen auf ihre Kinder von Mai 2020 bis Februar 2021 (von der ersten zur zweiten Coronawelle) – vor der Verfügbarkeit eines Impfstoffes für Kinder – entwickelte und welche Determinanten der elterlichen Impfintention es gibt.

## Methoden

### Design

Die Studie verwendet Daten der KUNO-Kids-Gesundheitsstudie [15]. Seit 2015 rekrutiert die KUNO-Kids-Studie in der KUNO Klinik St. Hedwig in Regensburg geborene Kinder und ihre Familien. Das Einzugsgebiet der Studie umfasst damit neben Stadt und Landkreis Regensburg auch die angrenzenden, überwiegend ländlich geprägten Landkreise. Die Region ist charakterisiert durch Bevölkerungszuwachs und eine sehr gute wirtschaftliche Lage. Die Coronapandemie war Anlass für zusätzliche Online-Befragungen, die im Mai 2020 und im Februar 2021 durchgeführt wurden [16, 17]. Diese Befragungen mit längsschnittlichem Design ergänzen die Datenbasis der laufenden Geburtskohortenstudie um Themen mit Pandemiebezug.

### Stichprobe

1296 Familien mit einem Kind im Alter zwischen 1,5 und 5,9 Jahren, die an der KUNO-Kids-Studie teilnehmen und zugestimmt hatten, für weitere Befragungen kontaktiert werden zu dürfen, wurden zur Teilnahme eingeladen. 612 Familien füllten den Online-Fragebogen zum ersten Erhebungszeitpunkt aus (entspricht einer Teilnahmerate von 50,1 %), 507 (82,8 % der Teilnehmer der ersten Befragung) auch den zum zweiten.

### Datenerfassung und -instrumente

Die Einladungen zur Teilnahme wurden jeweils per Brief versandt. Dort wurde ein individueller Code angegeben, über den die Teilnahme an den Online-Befragungen möglich war. Da es sich bei der Zielgruppe um junge Eltern handelte, wurde eine ausreichende Online-Affinität vorausgesetzt. Die Online-Befragung wurde mit Hilfe der Software qnome (qnome.eu) erstmals im Mai 2020, dann ein weiteres Mal im Februar 2021 durchgeführt. Zu diesen Zeitpunkten war jeweils die erste bzw. die zweite Welle der Coronapandemie am Abklingen. Eine COVID-19-Impfung war für Kinder in Deutschland noch nicht zugelassen.

#### Determinanten der COVID-19-Impfintention.

Befragt wurden die Teilnehmenden in beiden Online-Befragungen jeweils zu Corona-Infektionen (keine – mit milden Symptomen – mit schweren Symptomen), zu Angehörigen der Risikogruppe in ihrem Umfeld (ja – nein), zu Sorgen um die eigene Gesundheit und um die Gesundheit der Familie, zur Beurteilung der politischen Maßnahmen zur Eindämmung der Pandemie, zur regelmäßigen Informationssuche zu Themen der Coronapandemie (jeweils 4‑stufige Likert-Skala) und zur wahrgenommenen Kompetenz bzgl. Schutzmaßnahmen (6-stufige Likert-Skala). Die Auswahl der Items erfolgte in Abstimmung mit einer weiteren Kohortenstudie [17] und orientierte sich an der für Deutschland repräsentativen COSMO-Studie [18]. Diese Variablen mit Bezug zur Coronapandemie wurden ergänzt um Daten zur Soziodemografie und zur Gesundheitskompetenz, welche für alle teilnehmenden Familien der KUNO-Kids-Studie bei Einschluss in die Studie erfasst wurden. Soziodemografische Variablen umfassten Alter der Mutter, Migrationshintergrund, höchster Schulabschluss, Berufstätigkeit der Elternteile und Alter des Kindes. Gesundheitskompetenz wurde mit der Skala „Krankheitsbewältigung“ des Health Literacy Survey – EU [19] erfasst. Die Skala hat 16 Items zum Finden, Verstehen, Beurteilen und Anwenden von Gesundheitsinformationen, die auf einer 4‑stufigen Likert-Skala beantwortet werden (Cronbachs Alpha: 0,91). Es wird ein Mittelwert berechnet und in eine Metrik von 0–50 transformiert, wobei höhere Werte einer höheren Gesundheitskompetenz entsprechen.

#### COVID-19-Impfintention.

Die Frage zur COVID-19-Impfintention bezogen auf das Kind berücksichtigte, dass zum Zeitpunkt der beiden Befragungen noch kein Impfstoff für Kinder zugelassen war. Die Frage: „Wenn es einen wirksamen Impfstoff gegen COVID-19 geben würde, würden Sie Ihr Kind dann impfen lassen?“, konnte auf einer 3‑stufigen Antwortskala (ja – weiß nicht – nein) beantwortet werden. Für die regressionsanalytische Auswertung wurden die Antworten dichotomisiert (ja vs. weiß nicht/nein).

### Statistik

Für metrische Variablen wurden Mittelwert und Standardabweichung berechnet, für kategoriale Variablen Prozente. Die Veränderung in den Determinanten der Impfintention zwischen den beiden Erhebungszeitpunkten wurde mittels Wilcoxon-Tests bzw. T‑Tests für abhängige Stichproben untersucht. Wegen der geringen Anzahl an Vätern, die an den Befragungen teilgenommen hatten, konnte das Geschlecht nicht als möglicher Einflussfaktor analysiert werden. Deswegen wurde bei der Analyse der Determinanten der elterlichen COVID-19-Impfintention die Stichprobe auf solche Fälle beschränkt, in denen die Mutter an den Befragungen teilgenommen hat. Unter Anwendung einer prädiktiven Modellierungsstrategie [20] wurden in einem ersten Schritt für beide Erhebungszeitpunkte die möglichen Determinanten der Impfintention in univariablen logistischen Regressionsmodellen analysiert. Determinanten, die zu einem der beiden Erhebungszeitpunkte im univariablen Modell mit* p* < 0,20 mit der Kriteriumsvariable Impfintention assoziiert waren, wurden in einem zweiten Schritt in ein multivariables Modell eingeschlossen. Als Effektschätzer werden Odds Ratios (OR) mit 95 % Konfidenzintervallen (95 % KI) berichtet. Ergebnisse mit einem Alpha bis 0,05 wurden als statistisch signifikant betrachtet. Alle Analysen wurden mit IBM.SPSS Version 28.0 durchgeführt.

## Ergebnisse

507 Familien, für die Daten aus beiden Befragungen vorliegen, bilden die Gesamtheit für die folgenden Analysen. In Tab. 1 sind die Charakteristika der Stichprobe zusammengefasst. In 79 % der Familien verfügte mindestens ein Elternteil über die (Fach‑)Hochschulreife. Das Kind, auf das sich die Befragung und damit die elterliche Impfintention bezog, war im Mittel 3,4 Jahre alt.

**Table 1:** 

Soziodemografische Merkmale	M (SD) bzw. Anteil absolut (relativ)
*Alter des Kindes (Jahre)*^a^	3,4 (0,9)
*Alter der Mutter (Jahre)*^a^	36,4 (3,9)
*Schulabschluss*	
Mit (Fach‑)Hochschulreife	400 (79 %)
Nach 10 Schuljahren	96 (19 %)
Nach weniger als 10 Schuljahren	9 (2 %)
*Migrationshintergrund*	53 (10 %)
*Berufstätigkeit*	
Beide Elternteile	316 (62 %)
Ein Elternteil	159 (31 %)
Kein Elternteil	4 (1 %)

Wegen fehlender Werte in manchen Variablen addieren sich Prozentangaben nicht auf 100 %

*M* Mittelwert, *SD* Standardabweichung

^a^Zum Zeitpunkt der ersten Befragung

**Figure 1:**
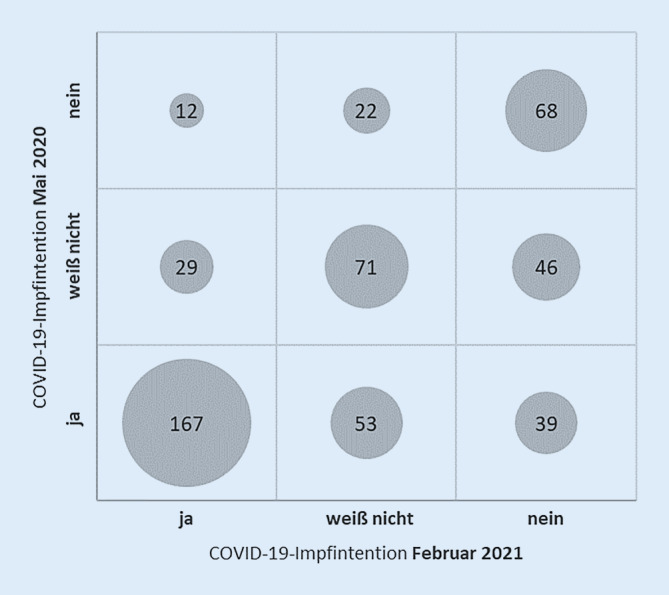

51 % der teilnehmenden Eltern (259/507) hatten im Mai 2020 die Intention, ihr Kind gegen COVID-19 impfen zu lassen; dieser Anteil reduzierte sich bis Februar 2021 auf 41 % (208/507). Abb. 1 veranschaulicht, in wie vielen Fällen die elterliche Impfintention gleich blieb bzw. sich veränderte. 167 Familien berichteten zu beiden Zeitpunkten von einer Impfintention („ja“), 71 antworteten zu beiden Zeitpunkten „weiß nicht“ und 68 hatten zu beiden Zeitpunkten keine Intention, das Kind impfen zu lassen. Unter allen Familien, die in der ersten Befragung von einer Impfintention bezogen auf das Kind berichteten („ja“), hatten 92 diese aber nicht mehr in der zweiten Befragung („weiß nicht“ oder „nein“). Umgekehrt lag in 41 Familien bei der ersten Befragung noch keine Impfintention vor („weiß nicht“ oder „nein“), bei der zweiten Befragung wurde dann aber von einer Impfintention berichtet („ja“).

Die Ausprägungen der verschiedenen möglichen Determinanten der COVID-19-Impfintention sind für die beiden Erhebungszeitpunkte in Tab. 2 dargestellt. Von der ersten zur zweiten Erhebung ging der Anteil derjenigen, die keinen Fall mit COVID-19-Erkrankung in der Familie oder im Freundeskreis hatten, von 77 % auf 35 % zurück; der Anteil derer, die im Umfeld einen Fall mit schwerer Symptomatik hatten, verdoppelte sich von 10 % auf 20 %. 96 % der Teilnehmenden in der zweiten Befragung im Vergleich zu 85 % in der ersten Befragung hatten nach eigener Einschätzung Familienangehörige oder Freunde, die zur Risikogruppe bzgl. einer Infektion mit dem Coronavirus gehörten. Auch die anderen erfassten Variablen spiegeln Veränderungen wider. Sorgen um die eigene Gesundheit oder die Gesundheit der Familie waren grundsätzlich nicht stark ausgeprägt, nahmen aber ebenso wie die eigene wahrgenommene Kompetenz im Umgang mit Schutzmaßnahmen zu. Das Vertrauen in die politischen Maßnahmen nahm dagegen im Mittel ab, das Gefühl, dass die Maßnahmen übertrieben seien, nahm zu. Mit Ausnahme der Variable zur regelmäßigen Informationssuche zur Coronapandemie waren die Unterschiede zwischen den beiden Erhebungszeitpunkten für alle Variablen statistisch signifikant.

**Table 2:** 

	Mai 2020	Februar 2021	*p*^a^
*COVID-19 bei Familienangehörigen/im Freundeskreis*			
Keine COVID-19-Fälle, *N* (%)	389 (76,7)	175 (34,5)	*–*
COVID-19-Fälle mit milder Symptomatik, *N* (%)	69 (13,6)	230 (45,5)	*–*
COVID-19-Fälle mit schwerer Symptomatik, *N* (%)	49 (9,7)	102 (20,1)	***<*** ***0,001***
*Familienangehörige/Freunde, die zur Risikogruppe gehören (ja), N (%)*	429 (84,6)	488 (96,3)	***<*** ***0,001***
*Sorgen um die eigene Gesundheit (0–4), M (SD)*	1,2 (0,9)	1,4 (0,8)	***<*** ***0,001***
*Sorgen um die Gesundheit der Familie (0–4), M (SD)*	2,1 (1,0)	2,2 (1,0)	***0,010***
*Wahrgenommene Kompetenz bzgl. Schutzmaßnahmen (0–6), M (SD)*	4,1 (1,3)	4,4 (1,3)	***<*** ***0,001***
*Vertrauen in Maßnahmen der Politik (0–4), M (SD)*	2,7 (0,9)	2,3 (1,0)	***<*** ***0,001***
*Wahrnehmung, dass politische Maßnahmen übertrieben sind (0–4), M (SD)*	0,7 (1,0)	0,9 (1,1)	***<*** ***0,001***
*Sich regelmäßig informieren über die Coronapandemie (0–4), M (SD)*	3,1 (0,9)	3,2 (1,0)	*0,350*

*M* Mittelwert, *SD* Standardabweichung

^a^Wilcoxon-Test für kategoriale Variablen, t‑Test für abhängige Stichproben für metrische Variablen

Tab. 3 zeigt die Ergebnisse der uni- und multivariablen Regressionsanalysen für die Kriteriumsvariable „COVID-19-Impfintention“. In die Analysen gingen nur die Fälle ein, in denen die Mütter den Fragebogen bearbeitet hatten (*N* = 546 bei der ersten Befragung, *N* = 457 bei den Befragungen). Die Analyse der Determinanten ergab für beide Befragungszeitpunkte ein sehr ähnliches Bild: Die Effektschätzer für die einzelnen Determinanten unterschieden sich nicht in der Richtung und nur geringfügig in ihrer Größe. Eine Ausnahme bildet die Variable zu COVID-19-Fällen in der Familie bzw. im Freundeskreis. Obwohl für beide Zeitpunkte die Assoziationen nicht statistisch signifikant waren, fällt auf, dass in der ersten Befragung jeweils mit der Kenntnis von leichten sowie schweren Erkrankungsfällen (im Vergleich zum Nichtkennen erkrankter Personen) eine höhere Impfintention bezogen auf das Kind einherging, während dies in der zweiten Befragung nur noch auf die Gruppe derer zutraf, die schwere Erkrankungsfälle kannten.

**Table 3:** 

	Mai 2020						Februar 2021					
	Univariabel			Multivariabel			Univariabel			Multivariabel		
	OR	95 % KI	*p*	OR	95 % KI	*p*	OR	95 % KI	*p*	OR	95 % KI	*p*
Alter des Kindes (Jahre)	1,12	0,91–1,38	*0,26*	–			1,05	0,85–1,3	*0,63*	–		
Alter der Mutter (Jahre)	1,04	0,99–1,09	*0,08*	1,02	0,97–1,07	*0,48*	1,03	0,98–1,09	*0,18*	1,02	0,97–1,08	*0,46*
Schulabschluss nach weniger als 10 Schuljahren^c^	0,48	0,09–2,44	*0,37*	0,92	0,16–5,28	*0,93*	0,31	0,04–2,62	*0,28*	–/–^a^		
Schulabschluss nach 10 Schuljahren^c^	Ref.						Ref.					
Schulabschluss mit Hochschulreife^c^	1,85	1,15–3,00	*0,01*	1,69	1,00–2,86	*0,052*	1,91	1,15–3,14	*0,01*	1,46	0,81–2,65	*0,21*
Migrationshintergrund^d^	1,06	0,59–1,90	*0,85*	–			1,15	0,64–2,10	*0,64*	–		
Gesundheitskompetenz^e^	1,02	0,99–1,04	*0,22*	1,00	0,97–1,03	*0,84*	1,05	1,03–1,08	*0,01*	**1,05**	**1,02–1,08**	***0,01***
Keine COVID-19-Fälle in Familie/Freundeskreis^b^	Ref.						Ref.					
COVID-19-Fälle mit milden Symptomen in Familie/Freundeskreis^b^	1,38	0,80–2,40	*0,25*	1,35	0,74–2,47	*0,32*	0,90	0,59–1,37	*0,61*	0,81	0,50–1,31	*0,40*
COVID-19-Fälle mit schweren Symptomen in Familie/Freundeskreis^b^	1,57	0,83–2,99	*0,17*	1,44	0,71–2,93	*0,31*	1,33	0,79–2,25	*0,28*	1,05	0,59–1,90	*0,86*
Familienmitglieder/Freunde, die zur Risikogruppe gehören^b^	0,63	0,37–1,09	*0,10*	**0,48**	**0,26–0,91**	***0,02***	0,32	0,11–0,95	*0,04*	**0,23**	**0,07–0,75**	***0,01***
Sorgen um die eigene Gesundheit (0–4)^b^	1,03	0,83–1,28	*0,79*	–			1,05	0,84–1,32	*0,65*	–		
Sorgen um die Gesundheit der Familie (0–4)^b^	1,17	0,97–1,41	*0,10*	1,15	0,93–1,43	*0,20*	1,10	0,91–1,33	*0,31*	1,14	0,91–1,43	*0,26*
Wahrgenommene Kompetenz bzgl. Schutzmaßnahmen (0–6)^b^	1,23	1,06–1,42	*0,01*	**1,19**	**1,01–1,39**	***0,03***	1,34	1,14–1,58	*0,01*	1,12	0,93–1,36	*0,24*
Vertrauen in Maßnahmen der Politik (0–4)^b^	1,55	1,25–1,93	*0,01*	1,22	0,93–1,61	*0,15*	1,68	1,37–2,06	*0,01*	1,10	0,82–1,46	*0,53*
Wahrnehmung, dass politische Maßnahmen übertrieben sind (0–4)^b^	0,60	0,49–0,74	*0,01*	**0,70**	**0,54–0,91**	***0,01***	0,56	0,45–0,70	*0,01*	**0,61**	**0,45–0,81**	***0,01***
Sich regelmäßig informieren über das Coronavirus (0–4)^b^	1,47	1,20–1,80	*0,01*	1,21	0,97–1,52	*0,09*	1,50	1,21–1,86	*0,01*	1,24	0,96–1,61	*0,09*

*N* = 457; Nagelkerkes R^2^: Mai 2020: 0,22; Februar 2021: 0,15

*OR* Odds Ratio, *KI* Konfidenzintervall, *p* Signifikanzwert, *Ref.* Referenzkategorie

^a^Keine gültigen Schätzer wegen unzureichender Variation in dieser Kategorie

^b^Zu beiden Survey-Zeitpunkten erfasst. Angaben aus dem ersten Survey wurden zur Prädiktion der Impfintention für den Mai 2020 genutzt, Daten des zweiten Survey für die Prädiktion der Impfintention für Februar 2021

^c^Höchster Schulabschluss des jeweils höher gebildeten Elternteils

^d^Migrationshintergrund: mindestens ein Elternteil außerhalb Deutschlands geboren

^e^Gesundheitskompetenz: erfasst durch die Krankheitsbewältigungsskala des HLS-EU

Signifikante positive Assoziationen zu mindestens einem der beiden Zeitpunkte gab es in den multivariablen Modellen für die Gesundheitskompetenz (2021: OR: 1,05, 95 % KI: 1,02–1,08) sowie die wahrgenommene Kompetenz bzgl. Schutzmaßnahmen gegen das Virus (2020: OR: 1,19, 95 % KI: 1,01–1,39). Die Einschätzung, dass Angehörige/Freunde zu einer Risikogruppe gehören (2020: OR: 0,48, 95 % KI: 0,26–0,91; 2021: OR: 0,23, 95 % KI: 0,07–0,75), sowie der Eindruck, dass die politischen Maßnahmen übertrieben seien (2020: OR: 0,70, 95 % KI: 0,54–0,91; 2021: OR: 0,61, 95 % KI: 0,45–0,81), gingen jeweils mit einer niedrigeren COVID-19-Impfintention bezogen auf das Kind einher. Die Varianzaufklärung für die beiden multivariablen Modelle war mit 22 % (2020) und 15 % (2021) gering.

## Diskussion

Nur rund die Hälfte der am Survey teilnehmenden Eltern intendierte nach der ersten Welle der Coronapandemie im Mai 2020 eine Impfung für ihre unter 6‑jährigen Kinder. Dieser Anteil reduzierte sich weiter bis zum nächsten Befragungszeitpunkt im Februar 2021 nach der zweiten Welle der Coronapandemie. Ein Grund für die beobachtete Abnahme der elterlichen Impfintention mag darin liegen, dass zum Zeitpunkt der ersten Befragung die Zulassung eines Impfstoffes noch nicht in Sicht war, während zum Zeitpunkt der zweiten Befragung die Impfkampagne für Erwachsene bereits seit ungefähr einem halben Jahr lief und zu dieser Zeit ein hohes Augenmerk auf Vor- und Nachteile sowie Nebenwirkungen der verschiedenen Impfstoffe gelegt wurde. Die Impfempfehlung, die sich an Erwachsene richtete, könnte nicht nur als Empfehlung für die Gruppe der Erwachsenen interpretiert worden sein, sondern auch in dem Sinne, dass eine Impfung für Kinder nicht empfehlenswert sei. Eine experimentelle Studie weist darauf hin, dass mit dem Aussprechen einer Empfehlung für eine Gruppe die Wahrnehmung einhergehe, dass eine andere Gruppe weniger betroffen sei [21]. Darüber hinaus war Anfang 2021 der Anteil an Personen, die bereits eine SARS-CoV-2-Infektion durchgemacht hatten, wesentlich höher als noch in den ersten Monaten der Pandemie. Eine durchgemachte Infektion ihres Kindes könnte von Eltern als Argument betrachtet worden sein, dass eine Impfung nicht mehr notwendig sei. Was das Muster der Determinanten der elterlichen Impfintention angeht, so war dieses über die beiden Erhebungszeitpunkte bemerkenswert stabil. Vor allem kompetenz- und einstellungsbezogene Variablen spielten eine Rolle.

Bei der Interpretation der Ergebnisse dieser Studie sind ihre Stärken und Schwächen zu berücksichtigen. Das längsschnittliche Design erlaubte es, die Entwicklung der COVID-19-Impfintention von Eltern von einer sehr frühen Phase der Pandemie bis zum Zeitpunkt, zu dem zumindest für Erwachsene eine Impfung verfügbar war, nachzuzeichnen. Die gewählte analytische Strategie macht es jedoch notwendig, Unterschiede in den Signifikanzen der Assoziationen zwischen den beiden Erhebungszeitpunkten nur zurückhaltend zu interpretieren: Mit diesen müssen nicht zwingend bedeutsame Unterschiede einhergehen. Die Teilnahmerate bei den Online-Befragungen war mit 50,1 % beim ersten und 41,5 % beim zweiten Survey gering; wie häufig bei Geburtskohortenstudien ist die Stichprobe der teilnehmenden Familien gekennzeichnet durch eine Überrepräsentation von Personen mit hohen Bildungsabschlüssen, Familien mit Migrationshintergrund sind dagegen kaum vertreten. Dieser Selektionsbias schränkt die externe Validität der Ergebnisse ein. Der Verlust von Teilnehmern von der ersten zur zweiten Befragung hatte dagegen keinen nennenswerten Einfluss auf die Ergebnisse. Sensitivitätsanalysen, in denen die Teilnehmer, die nur an der ersten Befragung teilgenommen hatten, mit denen, die an beiden Befragungen teilnahmen, verglichen wurden, zeigten keine relevanten Unterschiede bzgl. der Verteilung der elterlichen Impfintention.

Mit dem Fortschreiten der COVID-19-Impfkampagne und der Zulassung sowie Empfehlung der Impfung für Kinder in zahlreichen Ländern wurde eine Vielzahl von Studien durchgeführt, die Determinanten der elterlichen COVID-19-Impfintention untersuchen. Bislang gibt es zwei systematische Übersichtsarbeiten mit Metaanalysen, die die Daten aggregieren und einen Überblick zum Forschungsstand erlauben [14, 22]. Galanis und Kollegen schlossen 44 Studien mit insgesamt mehr als 300.000 Studienteilnehmern ein, die bis Dezember 2021 publiziert waren [14]. Alle Studien hatten ein querschnittliches Design. Die Qualität der Primärstudien wurde überwiegend als gut bewertet. Der Anteil von Eltern, die bereit waren, ihr Kind gegen COVID-19 impfen zu lassen, lag im Mittel bei 60 % und variierte von 26–92 %. Während die Ergebnisse zu soziodemografischen Determinanten der COVID-19-Impfintention uneinheitlich waren, zeigte sich vor allem für elterliche Einstellungen und Überzeugungen ein konsistenteres Bild. Die Arbeit von Chen et al. [22] wurde zu einem früheren Zeitpunkt fertiggestellt und schloss entsprechend weniger Primärstudien mit ein, kam aber, was die Prävalenz der elterlichen COVID-19-Impfintention bezogen auf das Kind angeht, zu vergleichbaren Ergebnissen. Auch hier wird die Bedeutung einstellungsbezogener Determinanten betont.

Einstellungen, die im Kontext einer COVID-19-Impfung von besonderem Interesse sind, beziehen sich auf Vertrauen in staatliche Institutionen und deren Glaubwürdigkeit. Die schiere Masse an verfügbarer Information, darunter auch Falschinformation bis hin zu Verschwörungstheorien, spiegelt sich im Begriff der „Infodemie“ wider [23] und macht es schwieriger, dass ausgewogene bzw. richtige Informationen zur Bevölkerung durchdringen. Bereits vor der Coronapandemie kam eine ländervergleichende Arbeit zum Schluss, dass es in Ländern mit einem hohen Maß an Zustimmung zu populistischen Parteien niedrigere Impfquoten gibt [24]. Unsere Studie zeigt, dass die Einschätzung, die politischen Maßnahmen seien übertrieben, mit einer niedrigeren Impfintention einherging. Das allgemeine Vertrauen in die politischen Maßnahmen zur Pandemiebekämpfung war allerdings in den multivariablen Modellen nicht mehr signifikant mit der Impfintention assoziiert.

Es ist eine Herausforderung für die Forschung, mit den Veränderungen, die sich im Zuge der Coronapandemie einstellen, Schritt zu halten, und notwendigerweise beziehen sich Ergebnisse empirischer Studien auf zwischenzeitlich vergangene Rahmenbedingungen. Neuere Daten aus einer deutschen Repräsentativbefragung von 1500 Eltern (September–Oktober 2021) zeigen, dass die Intention, das eigene Kind impfen zu lassen, stark mit dem Impfstatus der Eltern zusammenhängt [25]. So berichteten 55 % der geimpften Eltern, ihre Kinder unter 12 Jahren impfen lassen zu wollen, im Vergleich zu nur 9 % der ungeimpften Eltern. Die derzeit aktuellsten Daten stammen aus einer Erhebung der COSMO-Studie vom Juni 2022 [26]. Für die Altersgruppe von 0–4 Jahren wird eine elterliche COVID-19-Impfbereitschaft von 30 % angegeben. Wenn es um die COVID-19-Impfbereitschaft von Eltern bezogen auf ihre Kinder geht, gehören zu diesen veränderlichen Rahmenbedingungen nicht nur die aktuelle Situation bezüglich Zulassung von Impfstoffen und die jeweilige STIKO-Empfehlung, sondern auch die Einschätzung und die mediale Berichterstattung über die Bedrohlichkeit der COVID-19-Pandemie, die Langzeitfolgen einer COVID-19-Infektion oder die Nebenwirkungen einer COVID-19-Impfung sowie die aktuelle Organisation der Impfkampagne. Hier kann zum Beispiel entscheidend sein, wie einfach oder schwierig der Zugang zur Impfung ist, ob aktiv eingeladen wird oder welche Akteure und Institutionen des Versorgungssystems die Impfung durchführen dürfen. Darüber hinaus bleiben aber trotz variabler Kontextbedingungen einstellungsbezogene Faktoren für die Impfintention von Eltern entscheidend. Die insgesamt sehr geringen Unterschiede, die die vorliegende Studie in den Determinanten der Impfbereitschaft zwischen Mai 2020 und Februar 2021 fand, deuten darauf hin.

## Fazit

Die Intention, das eigene Kind gegen COVID-19 impfen zu lassen, war zu beiden Erhebungszeitpunkten nur mäßig ausgeprägt und nahm von der ersten zur zweiten Coronawelle noch weiter ab. Einstellungs- und kompetenzbezogene Determinanten standen zu beiden Zeitpunkten im Zusammenhang mit der Impfintention und könnten in einer künftigen Impfkampagne, die Eltern jüngerer Kinder adressiert, gezielt berücksichtigt werden. Strategien der Kommunikation und Öffentlichkeitsarbeit, die eine adäquate Risikowahrnehmung unterstützen sowie umfassend und orientiert an den individuellen Kompetenzen informieren, sollten zum Einsatz kommen.

## Supplementary Information




