# Supplementary material for: Parental intention to vaccinate their children against COVID-19
Source: Bundesgesundheitsblatt Gesundheitsforschung Gesundheitsschutz. 2022 Nov 9;65(12):1281–8. [Article in German] doi: 10.1007/s00103-022-03613-z (PMC9645347; doi:10.1007/s00103-022-03613-z)
Supplement: Supplementary file 1 [file 103_2022_3613_MOESM1_ESM.pdf]

Bundesgesundheitsblatt – Gesundheitsforschung – Gesundheitsschutz, Ausgabe 12/2022

Onlinematerial zum Beitrag:

COVID-19-Impfintention von Eltern bezogen auf ihre Kinder

Susanne Brandstetter[1,2], Maja Pawellek[1,2], Merle M. Böhmer[3,5], Angela Köninger[2,4], Michael Melter [1,2], Michael Kabesch[1,2], Christian Apfelbacher[2,5], KUNO Kids Study Group

[1] Kinderuniversitätsklinik Ostbayern (KUNO), Regensburg, Deutschland
[2] Wissenschafts- und Entwicklungscampus Regensburg (WECARE), Klinik St. Hedwig, Barmherzige Brüder, Regensburg, Deutschland
[3] Bayerisches Landesamt für Gesundheit und Lebensmittelsicherheit (LGL), München, Deutschland
[4] Klinik für Geburtshilfe und Frauenheilkunde der Universität Regensburg, Klinik St. Hedwig, Regensburg, Deutschland
[5] Institut für Sozialmedizin und Gesundheitssystemforschung, Otto von Guericke Universität Magdeburg, Magdeburg, Deutschland

Korrespondenzadresse:
Prof. Dr. Dr. Christian Apfelbacher
Medizinische Fakultät
Otto-von-Guericke-Universität Magdeburg
Institut für Sozialmedizin und Gesundheitssystemforschung
Leipziger Straße 44
39120 Magdeburg
Deutschland
christian.apfelbacher@med.ovgu.de

Inhalt

Tabelle: COVID-19-bezogene Items zur Impfintention und zu Determinanten der Impfintention

COVID-19-bezogene Items zur Impfintention und zu Determinanten der Impfintention

Thema und Item	Antwortmöglichkeit
COVID-19-Fälle im Familien-/Bekanntenkreis	
Gibt bzw. gab es in der Familie und dem Bekanntenkreis (inkl. des Freundeskreises des Kindes/der Kinder) mindestens eine nachgewiesene Corona-Erkrankung?	*nicht, dass ich wüsste – ja, aber nur mit leichten Symptomen – ja, mindestens eine Person ist/war ernsthaft erkrankt*
Familienmitglieder/Freunde, die zur Risikogruppe gehören	
Gehört jemand aus der Familie/dem Freundeskreis zu einer Risikogruppe bezüglich des Coronavirus?	*ja – nein*
Sorgen um die eigene Gesundheit	
Ich mache mir Sorgen um meine eigene Gesundheit.	*überhaupt nicht – ein wenig – mittelmäßig – ziemlich – sehr*
Sorgen um die Gesundheit der Familie	
Ich mache mir Sorgen um die Gesundheit meiner Angehörigen.	*überhaupt nicht – ein wenig – mittelmäßig – ziemlich – sehr*
Wahrgenommene Kompetenz bzgl. Schutzmaßnahmen	
Wie sicher oder unsicher fühlen Sie sich bei der Frage, welche Schutzmaßnahmen geeignet sind, um eine Infektion mit dem neuartigen Coronavirus zu vermeiden?	*0 (sehr unsicher) bis 6 (sehr sicher)*
Vertrauen in Maßnahmen der Politik	
Ich habe Vertrauen in die getroffenen Maßnahmen von Bund und Land.	*überhaupt nicht – ein wenig – mittelmäßig – ziemlich – sehr*
Wahrnehmung, dass politische Maßnahmen übertrieben sind	
Ich finde die verordneten Vorsichtsmaßnahmen übertrieben.	*überhaupt nicht – ein wenig – mittelmäßig – ziemlich – sehr*
Sich regelmäßig informieren über das Coronavirus	
Ich informiere mich regelmäßig über die aktuellen Entwicklungen in der Corona-Krise.	*überhaupt nicht – ein wenig – mittelmäßig – ziemlich – sehr*
Elterliche Impfintention	
Wenn es einen wirksamen Impfstoff gegen COVID-19 geben würde, würden Sie Ihr Kind dann impfen lassen?	*nein – ja – weiß nicht*
